# A264 IDENTIFYING CLINICAL PREDICTORS FOR SUCCESS OF EXCLUSIVE ENTERAL NUTRITION INDUCTION THERAPY IN PEDIATRIC CROHN DISEASE

**DOI:** 10.1093/jcag/gwad061.264

**Published:** 2024-02-14

**Authors:** R G Suarez Suarez, D G McClement, H Huynh, A Griffiths, A Shaikh, A Otley, K Jacobson, M Sherlock, D Mack, C Deslandres, W El-Matary, J deBruyn, T Walters, E Wine

**Affiliations:** Paediatrics, University of Alberta, Edmonton, AB, Canada; Paediatrics, University of Alberta, Edmonton, AB, Canada; Paediatrics, University of Alberta, Edmonton, AB, Canada; The Hospital for Sick Children Department of Paediatrics, Toronto, ON, Canada; The Hospital for Sick Children Department of Paediatrics, Toronto, ON, Canada; Dalhousie University, Halifax, NS, Canada; BC Children's Hospital, Vancouver, BC, Canada; McMaster University, Hamilton, ON, Canada; University of Ottawa, Ottawa, ON, Canada; Universite de Montreal, Montreal, QC, Canada; University of Manitoba, Winnipeg, MB, Canada; University of Calgary, Calgary, AB, Canada; The Hospital for Sick Children Department of Paediatrics, Toronto, ON, Canada; Paediatrics, University of Alberta, Edmonton, AB, Canada

## Abstract

**Background:**

Current treatments for IBD focus on reducing inflammation, mostly through suppression of the immune system. Exclusive enteral nutrition (EEN) is recognized as the first line therapy for mild-to-moderate luminal pediatric Crohn disease patients pCD. Although EEN is safe, as it does not suppress the immune system, it poses considerable challenges to patients, mostly due to palatability and monotony of the formula and treatment costs. Moreover, the efficacy of EEN varies greatly from patient to patient. Therefore, there is a need to distinguish between responders and non-responder patients.

**Aims:**

Identify clinical features associated with efficacy of EEN induction therapy and apply machine learning to build a classifier to identify EEN non-responders.

**Methods:**

The Canadian Children Inflammatory Bowel Disease Network prospectively enrolled and followed new onset pediatric IBD cases. Prospective data for 308 pCD with EEN as their first treatment for CD are available. Patients with weighted Pediatric CD Activity Indexes (wPCDAI) collected after at least 4 weeks on EEN were compiled into a dataset (n=108). For this analysis, treatment response was defined as a wPCDAI reduction of at least 12.5 points of a patient’s wPCDAI baseline score.

The dataset includes 26 features for each of the 108 pCD patients at time of diagnosis. This included blood test results (Hgb, ESR, CRP, Alb, Htc, Plt), Paris Classifications, height, and weight Z-scores, as well as wPCDAI, SES-CD (simple endoscopic score, CD), PGA (physician global assessment), and Mayo scores.

Odds ratios were calculated to determine whether any features were associated with response to EEN induction therapy. Then, the most relevant features were identified with regularization techniques and a machine learning classifier for predicting response to EEN was built.

**Results:**

Results showed that the appropriate subset of features to include for optimal accuracy over model simplicity are n=4 (PUCAI, PGA, SES-CD scores, and hematocrit). Also, an increase in PGA score (e.g., moving from “mild disease” to “moderate disease”) was associated with an increase in the chance of EEN failure (OR 3.1, 95% CI [1.7,5.8], p=0.0001). The best classifier for predicting EEN response was a random forest consisting of 20 decision trees. The classifier achieved an area under the ROC curve of 0.75 ± 0.07

**Conclusions:**

Our results suggest that it is possible to produce a classifier capable of predicting EEN clinical remission with an accuracy over 60%. Higher PGA, PUCAI, and SES-CD scores show potential for predicting patients less likely to respond to EEN induction. This research has the potential to provide better quality of life for children who live with IBD.

**Funding Agencies:**

CIHRIMAGINE SPOR Network , Women and Children's Health Research Institute

